# Development of overweight and obesity in children. Results of the KiGGS cohort

**DOI:** 10.17886/RKI-GBE-2018-030.2

**Published:** 2018-03-15

**Authors:** Anja Schienkiewitz, Stefan Damerow, Elvira Mauz, Felicitas Vogelgesang, Ronny Kuhnert, Angelika Schaffrath Rosario

**Affiliations:** Robert Koch Institute, Berlin, Department of Epidemiology and Health Monitoring

**Keywords:** BODY MASS INDEX, OVERWEIGHT, OBESITY, HEALTH MONITORING, KIGGS COHORT

## Background

In recent years, the prevalence of overweight and obesity among children in Germany has stabilised at a high level [1]. Children and adolescents with overweight or obesity have an increased risk that an excessive body weight will persist throughout childhood and adolescence until adulthood [2]. For Germany, there are only a few prospective studies available that have examined the development of overweight and obesity among children and young people [3-5].

With the completion of KiGGS Wave 2, measurements of body height and weight in a population-based cohort are once again available. This allows us to describe the progression of children and adolescents with and without overweight or obesity at the time of the initial examination (KiGGS baseline study, 2003-2006) over a period of eleven years. The focus of this article is overweight and obesity among children who were aged 2 to 6 years at the time of the KiGGS baseline study and the transition probabilities until they reach the ages of 12 to 17 years at the time of KiGGS Wave 2.

## Indicator and methodology

The KiGGS baseline interview and examination survey (2003-2006) included 17,641 children and adolescents aged 0 to 17 years, of which 4,820 were aged 2 to 6 years [6, 7]. In the second follow-up survey (KiGGS Wave 2, 2014-2017) 10,853 children and adolescents aged 10 to 31 years old participated again [8]. The present analyses are based on data from children and adolescents for which valid measurements on body height and weight were available both for the KiGGS baseline study at the age of 2 to 6 years as well as at the age of 12 to 17 years at the time of KiGGS Wave 2 (n=2,568 children and adolescents; n=1,311 girls, n=1,257 boys).

The body mass index (BMI, in kg/m^2^) was calculated from body weight and height. Normal weight (≤90th percentile, P90), overweight (>P90 to ≤P97) and obesity (>P97) were defined using the national German percentiles [9, 10]. Overweight is thus defined as overweight without obesity.

In the present article, the proportions (%, including 95% confidence intervals, 95% CI) of cohort participants with and without overweight or obesity at the time of participation in the KiGGS baseline study and KiGGS Wave 2 are reported. Furthermore, transition probabilities (%, 95% CI) for overweight and obesity within the observation period of eleven years are presented.

In the analyses, weighting factors were used to take into account possible bias due to drop-out and selective reparticipation [8].

**KiGGS Wave 2**

Second follow-up to the German Health Interview and Examination Survey for Children and Adolescents

**Data owner:** Robert Koch Institute

**Aim:** Providing reliable information on health status, health-related behaviour, living conditions, protective and risk factors, and health care among children, adolescents and young adults living in Germany, with the possibility of trend and longitudinal analyses

**Study design**: Combined cross-sectional and cohort study

**Cross-sectional study in KiGGS Wave 2**

**Age range:** 0-17 years

**Population:** Children and adolescents with permanent residence in Germany

**Sampling:** Samples from official residency registries - randomly selected children and adolescents from the 167 cities and municipalities covered by the KiGGS baseline study

**Sample size:** 15,023 participants

**KiGGS cohort study in KiGGS Wave 2**

**Age range:** 10-31 years

**Sampling:** Re-invitation of everyone who took part in the KiGGS baseline study and who was willing to participate in a follow-up

**Sample size:** 10,853 participants

**KiGGS survey waves**

► KiGGS baseline study (2003-2006), examination and interview survey
► KiGGS Wave 1 (2009-2012), interview survey
► KiGGS Wave 2 (2014-2017), examination and interview survey

More information is available at www.kiggs-studie.de/english

## Results

At the beginning of the KiGGS cohort study, 7% (95% CI: 6%-8%) of the study population aged 2 to 6 years were overweight and 3% (95% CI: 2%-4%) were obese. After eleven years of follow-up at the time of KiGGS Wave 2, the proportion of adolescents who presented with overweight (9%; 95% CI: 8%-11%) or obesity (8%; 95% CI: 7%-10%) was considerably higher.

The majority of the 2 to 6 year-old girls and boys without overweight or obesity at the KiGGS baseline study had no overweight or obesity in adolescence, either (86%, 95% CI: 84%-88%). 8% (95% CI: 7%-10%) of these children developed overweight and 5% (95% CI: 4%-7%) obesity. Of the 2 to 6 year-old children without obesity, 93% (95% CI: 91%-95%) continued without obesity in adolescence.

**Figure 1:**
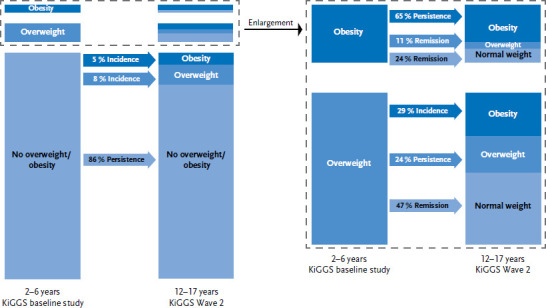
Development of overweight and obesity (n=1,311 girls, n=1,257 boys) Source: KiGGS baseline study (2003-2006), KiGGS Wave 2 (2014-2017)

Of the 2 to 6 year-old children with overweight, 24% (95% CI: 17%-33%) remained in this category after eleven years, 29% (95% CI: 20%-39%) changed to obesity and 47% (95% CI: 37%-57%) were no longer overweight or obese in adolescence. Of the children with obesity, 65% (95% CI: 47%-80%) remained obese, while 11% (95% CI: 5%-24%) changed to the category overweight and 24% (95% CI: 12%-42%) shifted into the normal weight category in adolescence (Figure 1).

## Discussion

The first results of the KiGGS cohort on the development of overweight and obesity over time indicate that the vast majority of children in kindergarten and preschool age within the study period are neither affected by overweight nor by obesity. However, the proportion of overweight and obese children in this young age group increases considerably during school age until adolescence. These changes can also be observed with prevalence estimates over time (trends) from the cross-sectional surveys of the KiGGS study [7, 8].

The first analyses of individual tracking of overweight and obesity in the KiGGS cohort also indicate that an excessive body weight among children in kindergarten and preschool age often remains until adolescence. Obesity in children in the age group 2 to 6 years is still present in more than half of them in adolescence; in addition, approximately one in four of the overweight children changes to the obesity category with increasing age. In summary, more than half of the 2 to 6 year-old children with overweight or obesity remain overweight or obese as adolescents. This result is also confirmed by a systematic review of cohort studies published so far [2]. In addition, in the KiGGS cohort approximately 1 in 12 children without overweight or obesity develops obesity from kindergarten and preschool age, and approximately 1 in 19 children develops obesity.

The results of the KiGGS cohort confirm that a higher body weight acquired at young ages often remains until adolescence. This illustrates the necessity to prevent the development of obesity both during kindergarten and school age. Less than half of the children manage to develop normal weight once they have acquired overweight or obesity. When interpreting these results, it must be taken into account that in longitudinal studies, such as the KiGGS cohort, the estimates are likely to be optimistic, since it cannot be excluded that adolescents with an excessively high body weight have participated less frequently in the study of KiGGS Wave 2 and that the proportions of persistent obesity or overweight are thus underestimated.

Due to different classification systems for overweight and obesity at the age of less than 18 years (percentiles) compared to adults starting at the age of 18 years (fixed cutoffs, e.g. 30 kg/m^2^ for obesity), the present analysis was restricted to the age group of 2 to 6 year-old children at the time of the KiGGS baseline study, so that they remained under 18 years of age in KiGGS Wave 2. In further analyses, it will be necessary to investigate the older age groups in more detail in order to describe the development of overweight and obesity through young adulthood. In addition, it is planned to identify determinants of different development states of overweight and obesity, and to describe the effects of overweight and obesity on future health behaviour.
