# Erratum: Development of overweight and obesity in children. Results of the KiGGS cohort

**DOI:** 10.25646/6798

**Published:** 2019-01-31

**Authors:** Anja Schienkiewitz, Stefan Damerow, Elvira Mauz, Felicitas Vogelgesang, Ronny Kuhnert, Angelika Schaffrath Rosario

Schienkiewitz A, Damerow S, Mauz E, Vogelgesang F, Kuhnert R et al. (2018)

Journal of Health Monitoring 3(1): 72-77.

DOI 10.17886/RKI-GBE-2018-030

In an earlier version of this article, the labels of the columns “overweight” and “obesity” were switched in Figure 1 on page 73.

The corrected version of the article is available at DOI 10.17886/RKI-GBE-2018-030.2

